# Toxin-Activating Stapled Peptides Discovered by Structural Analysis Were Identified as New Therapeutic Candidates That Trigger Antibacterial Activity against *Mycobacterium tuberculosis* in the *Mycobacterium smegmatis* Model

**DOI:** 10.3390/microorganisms9030568

**Published:** 2021-03-10

**Authors:** Sung-Min Kang, Heejo Moon, Sang-Woo Han, Byeong Wook Kim, Do-Hee Kim, Byeong Moon Kim, Bong-Jin Lee

**Affiliations:** 1 College of Pharmacy, Duksung Women’s University, Seoul 01369, Korea; smkang@duksung.ac.kr; 2 Department of Chemistry, College of Natural Sciences, Seoul National University, Seoul 08826, Korea; hj.moon@snu.ac.kr (H.M.); bwkim1324@snu.ac.kr (B.W.K.); 3 The Research Institute of Pharmaceutical Sciences, College of Pharmacy, Seoul National University, Seoul 08826, Korea; snu_hsw@snu.ac.kr; 4 College of Pharmacy, Jeju National University, Jeju 63243, Korea; doheekim@jejunu.ac.kr; 5 Interdisciplinary Graduate Program in Advanced Convergence Technology & Science, Jeju National University, Jeju 63243, Korea

**Keywords:** toxin-antitoxin system, structure-based drug discovery, antimicrobial candidate, stapled peptide

## Abstract

The structure-function relationships of toxin-antitoxin (TA) systems from *Mycobacterium tuberculosis* have prompted the development of novel and effective antimicrobial agents that selectively target this organism. The artificial activation of toxins by peptide inhibitors can lead to the growth arrest and eventual death of bacterial cells. Optimizing candidate peptides by hydrocarbon α-helix stapling based on structural information from the VapBC TA system and in vitro systematic validation led to **V26-SP-8**, a VapC26 activator of *M. tuberculosis*. This compound exhibited highly enhanced activity and cell permeability owing to the stabilizing helical propensity of the peptide. These characteristics will increase its efficacy against multidrug-resistant tuberculosis and extensively drug-resistant tuberculosis. Similar approaches utilizing structural and biochemical information for new antibiotic targets opens a new era for developing TB therapies.

## 1. Introduction

Bacterial toxin-antitoxin (TA) systems are modules that play an essential role in cell survival by acting as coupled regulators. Typically, toxins act as negative regulators through various mechanisms, such as causing RNA cleavage and cell membrane damage or inhibiting the synthesis of DNA gyrase and ATP [1,2]. Through this negative regulation, toxins can cause bacterial cell growth arrest and eventually cell death. In contrast, antitoxins serve as positive regulators of cell survival. Antitoxins bind toxins at the protein or gene level to neutralize their toxic activity. Therefore, to avoid toxic conditions and maintain the life cycle, the expression levels and interactions of toxins and antitoxins must be balanced. Since TA systems act as controllers of bacterial survival, they are recognized as promising antibacterial targets for the development of new antibacterial agents [3,4]. For instance, some approaches utilize peptides or peptide nucleic acid to targeting TA systems to exert an antibacterial effect [2,3].

Many TA systems exist in the *Mycobacterium tuberculosis* genome. Surprisingly, proteins belonging to the TA system account for more than 5% of all proteins in the H37Rv strain, which is a virulent *M. tuberculosis* strain [5]. The treatment of *M. tuberculosis* infection still requires long-term multidrug therapy owing to multidrug-resistant tuberculosis and extensively drug-resistant tuberculosis [6,7]. Therefore, new antimicrobial agents with mechanisms that are completely different from those of current antibiotics are needed. Studies of the TA system indicate that it is likely relevant to bacterial pathogenicity and infection. Its functional and regulatory roles in bacterial survival have been studied over the last 10 years and will hopefully lead to lasting cures [8,9].

A rational approach to exploit the TA system as a potential drug target is the artificial activation of toxins. A deeper understanding of the structure-function relationships in *M. tuberculosis* TA systems has enabled scientists to devise new strategies for developing effective drug candidates targeting *M. tuberculosis*. In this strategy, peptides or small molecules are used as inhibitors of intermolecular protein–protein (TA) interactions. Under normal circumstances, toxins do not exert a toxic effect because antitoxin blocks the toxin active site, forming a complex with the toxin. However, when a complex binding inhibitor is added to the TA complex, it competes with one of two binding partners (toxin or antitoxin). Thus, the TA complex is broken, and free toxin is released from the TA complex [10,11].

In a previous study, peptide inhibitors designed to mimic the binding interface of a TA complex based on structural and biochemical information of the *M. tuberculosis* VapBC26 complex exhibited TA complex-disrupting activity in vitro [12]. Three peptides that mimic the binding interface were selected on the basis of the crystal structure of the VapBC26 complex (*Rv0581-Rv0582*, PDB code 5 × 3T) [12]. The ribonuclease (RNase) activity of VapC26 (*Rv0582*) was not detected when VapC26 was bound to its cognate antitoxin, VapB26 (*Rv0581*), in the TA complex. In contrast, those peptides mimicking the binding interface promptly regenerated the RNase activity of VapC26 by successfully disrupting the VapBC26 TA complex. In a previous study, the expression of the toxin *Rv0582* inhibited *Mycobacterium smegmatis* cell growth [13]. Furthermore, TA systems are not present in eukaryotic cells, and bacterial pathogens are characterized by the prevalence of TA systems. In a recent study on the VapBC30 system in *M. tuberculosis*, a structurally modified peptide exerted bactericidal activity by disturbing the interaction within the VapBC30 complex [14]. Accordingly, the artificial activation of toxins appears to be an ideal strategy for the development of novel antibacterial drugs that function via this mechanism, which is found only in bacteria [10,11,15,16,17].

In this study, an initial candidate peptide identified in a previous study was varied through the site-directed mutagenesis of every residue into alanine. Using the alanine scans, we ranked the amino acid residues in the peptides according to their influence on the inhibitory activity of the initial peptide. The residues with the least influence on activity were selected as eligible residues for stapling modification. The synthesized stapled peptide **V26-SP-8** was characterized as the final candidate through circular dichroism (CD) spectroscopy and efficacy tests including in vitro RNase competition assays and binding affinity measurements via isothermal calorimetry (ITC). Moreover, successful membrane penetration and *M. smegmatis* cell killing by the inhibitor peptide were verified by confocal microscopy. Because *M. smegmatis* and *M. tuberculosis* have similar characteristics, *M. smegmatis* has been used as a model organism for the study of *M. tuberculosis* [18,19]. The efficacies of the final candidates were validated by a cell growth inhibition assay.

## 2. Materials and Methods

### 2.1. Protein Preparation

The VapC26 (*Rv0582*) and VapB26 (*Rv0581*) genes were amplified by a polymerase chain reaction. The cloned plasmids were transformed into *Escherichia coli* Rosetta2(DE3) pLysS competent cells. The transformed cells were cultured, and protein was extracted via ultrasonication and purification processes. Detailed procedures used for purifying the VapBC26 complex and VapC26 have been previously reported [12].

### 2.2. Peptide Synthesis

The stapled peptides were synthesized using Fmoc-protected amino acids and MBHA rink amide resin according to previously reported methods [20,21]. The synthesis was conducted using a slightly modified procedure (Figure S1 and Table S1). The peptides were prepared using Fmoc chemistry on Rink Amide MBHA Resin (BeadTech, Ansan, Korea) with a loading capacity of 0.41 mmol/g. All syntheses were conducted at an 82 μmol scale. The dry resin was swelled with N,N-dimethylformamide (DMF) for 2 h before use. The Fmoc protecting group was removed by treatment with 20% piperidine in DMF (2 × 10 min). After Fmoc deprotection, the resin was washed with DMF (×3), methanol (MeOH, ×3), dichloromethane (DCM, ×3), and N-methyl-2-pyrrolidone (NMP, ×3). Both natural and nonnatural amino acids were coupled for 2 h using 4 equiv. of (1-cyano-2-ethoxy-2-oxoethylidenaminooxy) dimethylamino-morpholino-carbenium hexafluorophosphate as an activating agent, 4 equiv. of Fmoc-protected amino acid, and 8 equiv. of diisopropylethylamine in NMP. After thorough washing with DMF (×9), the completeness of the coupling reaction was confirmed by the Kaiser test. Ring-closing metathesis of resin-bound *N*-Fmoc and side-chain-protected peptides was performed using 20 mol% Grubbs catalyst in 1,2-dichloroethane for 2 h at room temperature under an Ar atmosphere. After washing with DCM (×2), MeOH (×2), and DCM (×2), the reaction was repeated two more times. After Fmoc removal, *N*-terminus modification of the peptides with either an acetyl group or fluorescein isothiocyanate (FITC) was performed according to the following conditions: (i) acetylation: acetic anhydride (30 equiv.) and diisopropylethylamine (60 equiv.) in NMP for 45 min at room temperature, or (ii) FITC (1.5 equiv.) in pyridine/DMF/DCM (*v*/*v*/*v* = 12/7/5) overnight at room temperature. The resin was washed with DMF (×3), MeOH (×3), and DCM (×3) and dried under vacuum overnight. The peptide was deprotected and cleaved from the resin by treatment with a mixture of trifluoroacetic acid (TFA)/triisopropylsilane/water (*v*/*v*/*v* = 95/2.5/2.5) for 2 h. The volatile components were then removed under reduced pressure. The crude mixture was dissolved in a 1:1 mixture of acetonitrile and water and filtered through a 0.45 μm syringe filter. The desired product was purified by a preparative HPLC system (Agilent Technology, 1260 Infinity, Santa Clara, CA, USA; solvent system: MeCN:H_2_O with 0.1% TFA; 0–40 min, 5–100% MeCN; 40–55 min, 100% MeCN; 55–60 min, 100–5% MeCN; flow rate: 10.0 mL/min; column: Zorbax C18 column, 5 μm, 80 Å, 21.2 × 150 mm). The purified peptide was characterized using an analytical HPLC system (Agilent Technology, 1260 Infinity, Santa Clara, CA, USA; solvent system: MeCN:H_2_O with 0.1% TFA; 0–5 min, 5–50% MeCN; 5–7 min, 50–100% MeCN; 7–15 min, 100% MeCN; 15–17 min, 100–5% MeCN; flow rate: 1.0 mL/min; column: Zorbax C18 column, 2.7 μm, 120 Å, 4.6 × 50 mm) and an ultrahigh resolution ESI Q-TOF mass spectrometer (Bruker, Billerica, MA, USA) at the Organic Chemistry Research Center of Sogang University (Korea). For the final candidate **V26-SP-8**, the stapling technique was applied between the first aspartate and the eighth arginine. The final purity of **V26-SP-8** was greater than 95%.

### 2.3. In Vitro Ribonuclease Assay

The ribonuclease activity measured the release of VapC26 from VapBC26 by the peptides. This assay was performed with an RNase Alert Kit (IDT, Coralville, IA, USA). In this assay, a fluorophore is covalently attached to the end of a synthetic RNA strand and quenched by a quencher at the other end of the synthetic RNA. If the synthetic RNA containing a fluorophore-quencher pair interacts with ribonuclease, the synthetic RNA is cleaved, and fluorescence is detected. The released fluorophores emit fluorescence at 520 nm upon excitation at 490 nm. The resulting fluorescence (in relative florescence units) was detected with a SpectraMax Gemini XS microplate reader (Molecular Devices, San Jose, CA, USA). Various concentrations of peptides (5, 10, 20, and 100 μM) and 100 μM VapBC26 complex were used. The protein complex does not itself fluoresce. The antitoxin blocks the toxin’s action, but if a peptide binds, the toxin is released from the complex, and the resulting fluorescence can be monitored.

### 2.4. CD Spectroscopy

CD measurements were conducted at 20 °C using a Chirascan Plus spectropolarimeter (Applied Photophysics, Leatherhead, UK) in a cuvette with a 1 mm path length. The peptide samples were prepared at 50 μM in buffer consisting of 10 mM potassium phosphate (pH 7) and 100 mM ammonium sulfate. CD scans were taken from 180 nm to 260 nm at a speed of 100 nm/min with a 1 nm bandwidth. The helicity of each peptide was calculated by CDNN software [22].

### 2.5. Isothermal Titration Calorimetry (ITC) Measurements

ITC experiments were performed with a MicroCal 200 (GE Healthcare, Chicago, IL, USA) at 25 °C. The protein and peptide were prepared in buffer consisting of 20 mM MES (pH 6) and 50 mM NaCl. The ITC experiments were performed with the protein solution (10 μM, 320 μL) in the cell and the peptide solution (600 μM) as the injected titrant. A total dataset of 19 injections made at 150 s intervals was collected. MicroCal Origin software was used for curve fitting to calculate the binding affinity (*K_d_*), enthalpy of binding (Δ*H*), entropy of binding (Δ*S*) and stoichiometry (*n*). The raw data were fitted by one-site binding. The Gibbs free energy (Δ*G*) was calculated using the standard equation Δ*G* = Δ*H* − TΔ*S*. These assays were performed using a procedure similar to that used in a previous study [12]. ITC experiments were also performed in the absence of proteins under the same experimental conditions, and no thermal changes were observed in this control experiment.

### 2.6. NMR Titration of VapB26 Antitoxin with Peptide

To understand the structural transition occurring in VapB26 upon peptide binding, NMR titration was conducted based on the backbone assignment of VapB26 [12]. The concentration of VapB26 in the three 2D ^1^H, ^15^N-HSQC spectra was 0.4 mM, and the peptide concentration varied from 0 to 0.08 mM (20% of the protein concentration). The samples were prepared in buffer containing 20 mM MES (pH 6), 50 mM NaCl, and 10% (*v*/*v*) D_2_O.

### 2.7. Cloning and Expression of TA Protein in M. smegmatis

The genes encoding *M. tuberculosis* (strain H37Rv) VapB26 (*rv0581*) and VapC26 (*rv0582*) were amplified using primers Rv0581-BHI_F, Rv0581-HindIII_r, Rv0582-Ndel_F, and Rv0582-EcoRV-r (Table S2) and were cloned into the pYUBDuet expression vector (Addgene, Watertown, MA, USA). This produced VapB26 with an N-terminal hexa-histidine tag and VapC26. The vector harboring *rv0581* and *rv0582* was transformed into *E. coli* Top10 competent cells (Thermo Fisher Scientific, Waltham, MA, USA). The plasmid extracted from transformed Top10 cells was electroporated into *M. smegmatis* mc^2^155. Preparation of the *M. smegmatis* competent cells and the electroporation were conducted according to reported protocols (Bio-Rad; Hercules, CA, USA, bio-rad.com/webroot/web/pdf/lsr/literature/Bulletin_1360.pdf, accessed on 10 March 2021). *M. smegmatis* mc^2^155 harboring *rv0581* and *rv0582* were grown at 37 °C and 100 rpm in Middlebrook 7H9 liquid culture medium supplemented with 10% albumin-dextrose-saline, 0.5% glycerol and 0.1% Tween 80. Hygromycin (Hyg, 50 μg/mL) was added to the *M. smegmatis* culture. When the OD_600_ of the culture reached 0.5, expression was induced by the addition of 0.5 mM isopropyl 1-thio-D-galactopyranoside, and the cells were grown at 37 °C for an additional 24 h.

### 2.8. Confocal Microscopy

Confocal microscopy was used to assess the permeability of *M. smegmatis* using FITC-labeled peptides. Cultures of *M. smegmatis* grown in complete 7H9 medium to mid-logarithmic phase (OD_600_ 0.3–0.5) were diluted 5-fold with Muller-Hinton broth (BD Biosciences, Franklin Lakes, NJ, USA). Diluted cell suspensions were aliquoted into Eppendorf tubes and treated with 10 μM peptide. After 1 h of incubation at 37 °C and 100 rpm, the cell suspensions were washed with PBS. If successful internalization of FITC-labeled peptides is achieved, then fluorescence will be detected in the images. Surface-bound peptides and extracellular peptides were degraded by trypsin, and thus remained fluorescence signals in the images indicate the indeed internalization of peptide. Images were acquired on a TCS8 confocal scope (Leica Microsystems, Wetzlar, Germany) with a 63× oil immersion objective.

### 2.9. Flow Cytometry

Flow cytometry was used to confirm the permeability of the peptides into *M. smegmatis*. The samples prepared for permeability assessment were prepared in the same manner as those for confocal imaging and staining with PI-phycoerythrin. The flow rate was 100 μL/min, and 10,000 events were collected. If successful internalization of FITC-labeled peptides is achieved, the resulting peaks are shifted or regenerated from the initial peak. All flow cytometry analyses were performed on a FACSLyric (BD Biosciences, Franklin Lakes, NJ, USA), and the data were analyzed using FlowJo software (TreeStar, Woodburn, OR, USA).

### 2.10. In Vitro Cell Growth Assay

An in vitro cell growth assay of recombinant *M. smegmatis* was carried out to assess the susceptibility of VapBC26-expressing *M. smegmatis* cells to the peptides. *M. smegmatis* cells overexpressing VapBC26 were grown at 37 °C in 7H9/ADC containing 50 μg/mL Hyg until reaching an OD_600_ of 1.0. The bacterial suspensions were diluted to an OD_600_ of 0.1 and dispensed into each well of a 96-well cell culture plate (300 μL/well). Stock peptide solutions were diluted from 25 μM to 6.25 μM by two-fold serial dilution in 7H9 broth and subsequently added to the wells containing the bacterial suspensions. The plate was incubated for 1 day at 37 °C; subsequently, the OD_600_ was monitored every other day for 7 days using a multireader (Molecular Devices, SpectraMax M5, San Jose, CA, USA). Each assay included control cells that did not contain any peptide derivatives. Error bars represent the standard error of the mean of three biological replicates.

## 3. Results and Discussion

### 3.1. Design of Stapled Peptides Based on Alanine Scanning of Linear Peptides

**Figure 1:**
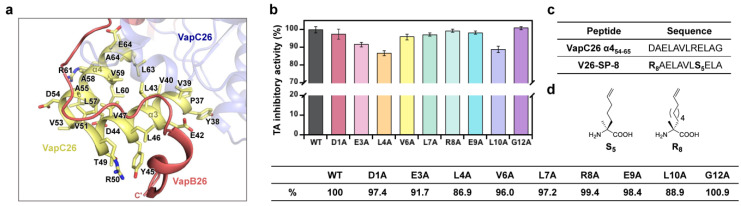
(**a**) X-ray crystal structure of the VapBC26 complex. The α-helical segments that are involved in the VapB26:VapC26 interaction are highlighted as follows: a binding interface in VapB26 (red) and VapC26 α3_37–52_ and α4_54–65_ (yellow). (**b**) Alanine scanning results of VapBC26-based linear peptides (VapC26 α4_54–65_). The activity was normalized to that of the original peptides. The concentration of each peptide was 10 μM. Data are presented as the mean ± SD of three independent replicates. Average values are additionally denoted in the table. (**c**) Sequences of VapC26 α4_54–65_ and its derivative **V26-SP-8**. (**d**) Chemical structures of olefin-bearing noncanonical amino acids that were used for peptide stapling.

As previously published by our group, the α-helical fragments at the binding region of VapC26 and VapB26 act as inhibitors of the corresponding TA protein–protein interaction [12]. In the interaction network between VapB26 and VapC26, the α4 and α5 helices of VapC26 are the main contributors to the interaction between VapB26 and VapC26 (Figure 1a). Because an α4-mimicking peptide (VapC26 α4_54–65_) showed the most potent complex disruption activity, we performed alanine scanning to verify the contribution of each amino acid residue to the inhibitory activity and identified reasonable positions of nonnatural amino acids for peptide stapling (Figure 1b). We added each alanine-substituted peptide to the VapBC26 complex and evaluated whether the peptide induced the ribonuclease activity of VapC26 in an in vitro RNase assay. For the VapC26 α4_54–65_ peptide, D1A and R8A did not result in a noticeable change in TA complex dissociation activity (<5% decrease from that of the wild type). Thus, the positions for olefin-bearing residues were determined according to the following criteria: (i) less than 5% change in inhibitory activity when substituted, and (ii) the two locations were at *i* and *i* + 3, 4 or 7 to align these residues to the same α-helical face (Figure 1c,d). Based on these results, we determined suitable stapling positions for designing the rational synthetic target **V26-SP-8**, which showed the best activity among the stapled peptides.

### 3.2. In Vitro RNase Assay of Stapled Peptides

**Figure 2:**
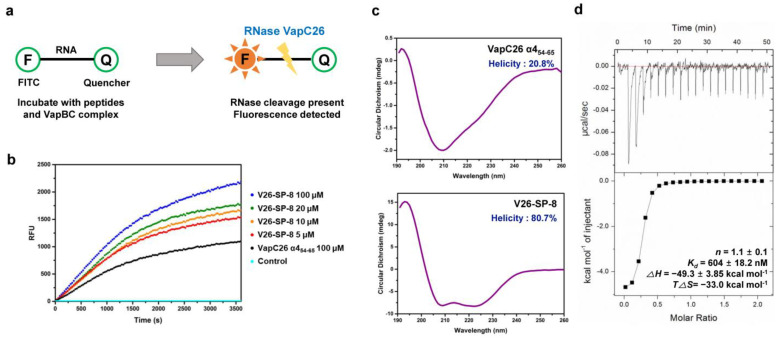
In vitro assay data for stapled peptides. (**a**) Schematic diagram of the in vitro ribonuclease activity assay. (**b**) In vitro ribonuclease activity assays of **V26-SP-8** and VapC26 α4_54–65_. The data shown are representative of three independent experiments. (**c**) CD spectra of VapC26 α4_54–65_ and **V26-SP-8**. The data shown are the averages of three independent scans. (**d**) ITC assay of the interaction between **V26-SP-8** and VapB26. Similar results were obtained in two independent experiments.

Based on the alanine scanning results, **V26-SP-8** was synthesized through solid-phase peptide synthesis at greater than 95% purity (Figure S1 and Table S1). We then conducted an in vitro RNase assay to investigate its ability to disrupt the interactions in the VapBC26 complex. In this experiment, the inhibitory peptide released the VapC26 toxin from the VapB26 antitoxin, leading to the restoration of VapC26 RNase activity. Thereafter, the substrate RNA, which operates as a connection between a fluorophore and a quencher, was cleaved. This process increases the fluorescence signal in proportion to the quantity of free VapC26 molecules (Figure 2a). **V26-SP-8** mimics the antitoxin-binding region of VapC26 and is designed to bind to VapB26. Therefore, **V26-SP-8** binds to VapB26 in competition with VapC26, thereby exposing the VapC26 active site and inducing the digestion of bacterial rRNA. In this way, our stapled peptide served as an antibacterial agent against VapBC26-expressing *M. tuberculosis*. The concentration dependence of **V26-SP-8** was also confirmed (Figure 2b). The results showed that **V26-SP-8** was more active than the linear peptide VapC26 α4_54–65_.

### 3.3. Helical Propensities of Linear and Stapled Peptides

The α-helical propensities of the stapled peptide **V26-SP-8** and its linear counterpart VapC26 α4_54–65_ were evaluated by CD spectroscopy. While a highly negative peak at approximately 210 nm appeared for VapC26 α4_54–65_, indicating a typical random coil pattern, **V26-SP-8** showed two obvious negative peaks at 208 and 222 nm, representing an α-helical structure in aqueous media (Figure 2c). The helical propensity of **V26-SP-8** was dramatically increased to 80.7% compared with 20.8% of the linear counterpart VapC26 α4_54–65_. Therefore, this result demonstrates the effectiveness of the peptide stapling strategy. This result might indicate that there is further room for structural optimization of the stapled peptide.

### 3.4. ITC Assay and NMR Titration of V26-SP-8 against the VapB26 Antitoxin

**Figure 3:**
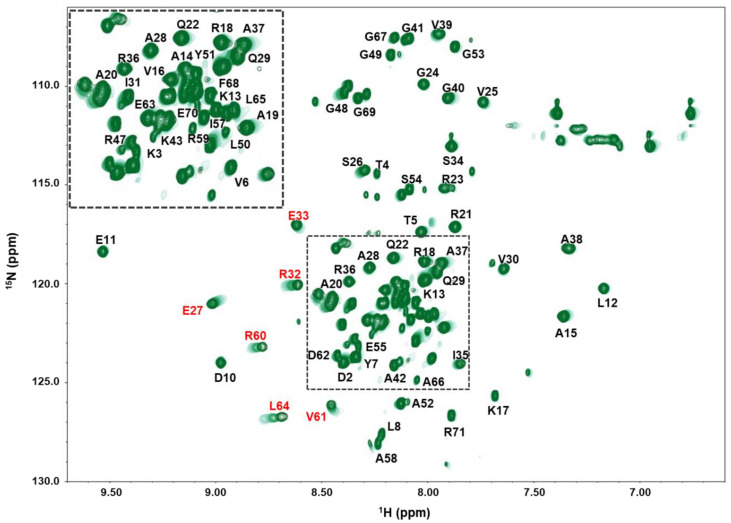
NMR titration of VapB26 with **V26-SP-8**. The residues in VapB26 showing notable CSP are marked in red.

An ITC assay was conducted to measure the binding affinity of **V26-SP-8** to the VapB26 antitoxin. The measured dissociation constant (*K_d_*) of VapB26 with **V26-SP-8** was 604 ± 18.2 nM (Figure 2d). In the NMR titration of V26-SP-8 into ^15^N-labeled VapB26 antitoxin^7^, the peaks of the VapB26 residues on the toxin-binding region exhibited chemical shift changes (Figure 3). Residues belonging to the α3 helix (R60, V61 and L64) showed a peak shift. Some residues on the α2 helix that belong to the DNA binding region (E27, R32 and E33) also exhibited changes in their chemical shifts. These results demonstrate that the binding of **V26-SP-8** to the VapB26 antitoxin interferes with the formation of the VapBC26 complex by binding to the α3 helix, which is the VapC26 binding site. **V26-SP-8** binding also affects the DNA binding domain of VapB26; this interferes with normal transcription and might adversely affect bacterial survival. Identification of the residues required for the interaction between VapB26 and **V26-SP-8** will help elucidate the mechanism of VapBC26 complex disruption.

### 3.5. Evaluation of the Cell Permeability and Antibacterial Activity of V26-SP-8

**Figure 4:**
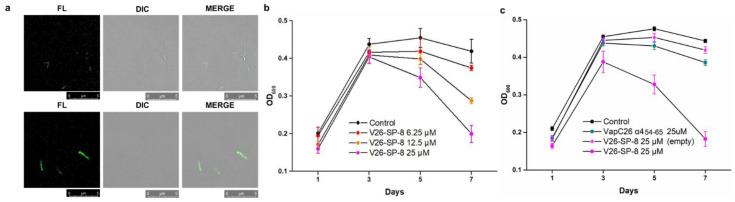
Confocal laser scanning microscopy detection of cell-penetrating peptides in living cells and the growth assay. Fluorescein-only (left) and brightfield images of *M. smegmatis* (middle) and overlaid images (right) are presented. (**a**) Confocal images of VapC26 α4_54–65_ (upper) and **V26-SP-8** (lower). Similar results were obtained in two independent experiments. (**b**,**c**) Error bars represent the standard error of the mean of three biological replicates. (**b**) *M. smegmatis* growth assay with **V26-SP-8** at different concentrations. (**c**) Growth assay including empty vector harboring *M. smegmatis* using **V26-SP-8** and VapC26 α4_54–65_ at 25 μM.

To examine whether the linear and stapled peptides penetrate the *M. smegmatis* cell membrane, we carried out confocal microscopy studies using FITC-labeled **V26-SP-8** and VapC26 α4_54–65_ in VapBC26-harboring *M. smegmatis* (Figure 4a). In the case of VapC26 α4_54–65_, the fluorescence signal was barely observed inside the cells, implicating its poor ability to penetrate the bacterial membrane. According to the CD spectroscopy results, the linear peptide did not form a helical structure in aqueous media, which might have caused the low cell permeability. In contrast, **FITC-V26-SP-8** was markedly taken up by *M. smegmatis*, which may be correlated with the helical propensity of the peptide. The flow cytometry data confirmed the enhanced cellular uptake of the stapled peptide compared with the linear peptide (Figure S2). As a result, we concluded that the stapling technique successfully increased the cell membrane penetration and localization of peptide in the cell. *M. smegmatis* mc^2^155 cell growth assays demonstrated the ability of **V26-SP-8** to arrest bacterial growth (Figure 4b). When *M. smegmatis* mc^2^155 cells were treated with **V26-SP-8**, cell growth was substantially decreased compared with that of the control cells, indicating the bactericidal activity of the structure-based designed peptide. VapC26 α4_54–65_ had only a slight effect on cell growth, and **V26-SP-8** had no effect on *M. smegmatis* mc2155 cells transformed with empty vector (Figure 4c). Successful **V26-SP-8** internalization was verified in the confocal images, demonstrating that the bactericidal effect occurred inside the bacterial cells. These data indicate the significant promise of **V26-SP-8** in the field of antibacterial drug discovery. In particular, because TA systems are prevalent in bacterial pathogens but are not present in eukaryotic cells, these systems are attractive drug targets that may provide high specificity and low side effects.

## 4. Conclusive Discussion

In this study, **V26-SP-8** was designed and produced using structural and biochemical information of the VapBC26 TA system from *M. tuberculosis* as a candidate for new antibacterial applications. We synthesized several stapled peptides that were based on VapC26 α4_54–65_. **V26-SP-8** was selected for focused study based on its initial in vitro activity (Figure S3). A systematic approach using stapled peptides that are based on the structural and biochemical information of TA systems will contribute to the further discovery of antibiotics that can artificially activate toxins by disrupting TA systems. Recent studies have applied peptides and peptide nucleic acids to disrupt TA systems as an antibacterial target. Although TA systems are prevalent in bacterial pathogens, they are not found in eukaryotic cells. This might increase the pathogen specificity of compounds that are based on TA systems, with lower side effects in humans. However, there is a concern that the moderation on the artificial activation of toxins might induce the formation of persister or dormant cells, thereby contributing to chronic infection. Thus, it is needed to develop a strategy that resuscitate dormant cells, rendering them susceptible to antibiotic drugs. Future studies must systemically validate the effectiveness of diverse antibiotic strategies that utilize pathogen TA systems. These approaches will aid in exploring putative antibacterial targets and new mechanisms that are different from the antibiotics used currently for TB treatment.

## Data Availability

Not applicable.

## Supplementary Materials

The following are available online at https://www.mdpi.com/2076-2607/9/3/568/s1: Figure S1: HPLC chromatogram of the purified peptides; Figure S2: Flow cytometry data; Figure S3: Data of the stapled peptides; Table S1: HRMS analysis of peptides; Table S2: Primers used for cloning.
