# Supplementary material for: Toxin-Activating Stapled Peptides Discovered by Structural Analysis Were Identified as New Therapeutic Candidates That Trigger Antibacterial Activity against Mycobacterium tuberculosis in the Mycobacterium smegmatis Model
Source: Microorganisms. 2021 Mar 10;9(3):568. doi: 10.3390/microorganisms9030568 (PMC8000039; doi:10.3390/microorganisms9030568)
Supplement: Supplementary file 1 [file microorganisms-09-00568-s001.pdf]

Toxin-Activating Stapled Peptides Discovered by Structural Analysis were Identified as New Therapeutic Candidates that Trigger Antibacterial Activity against *Mycobacterium Tuberculosis*

Sung-Min Kang[1,†], Heejo Moon[2,†], Sang-Woo Han[1], Byeong Wook Kim[2], Do-Hee Kim[3,4], Byeong Moon Kim[2,*] and Bong-Jin Lee[1,*]

[1]The Research Institute of Pharmaceutical Sciences, College of Pharmacy, Seoul National University, Seoul 08826, Republic of Korea

[2]Department of Chemistry, College of Natural Sciences, Seoul National University, Seoul 08826, Republic of Korea

[3]College of Pharmacy, Jeju National University, Jeju 63243, Republic of Korea

[4]Interdisciplinary Graduate Program in Advanced Convergence Technology & Science, Jeju National University, Jeju 63243, Republic of Korea

[†]These authors contributed equally to this work.

***Corresponding Authors**

E-mail: lbj@nmr.snu.ac.kr (B.J.L); +82-2-880-7868

E-mail: kimbm@snu.ac.kr (B.M.K); +82-2-880-6634

Contents

Figure S1: HPLC chromatogram of the purified peptides.

Figure S2: Flow cytometry data.

Figure S3: Data of the stapled peptides.

Table S1: HRMS analysis of peptides.

Table S2: Primers used for cloning.

V26-Pep-8 (>95% purity)

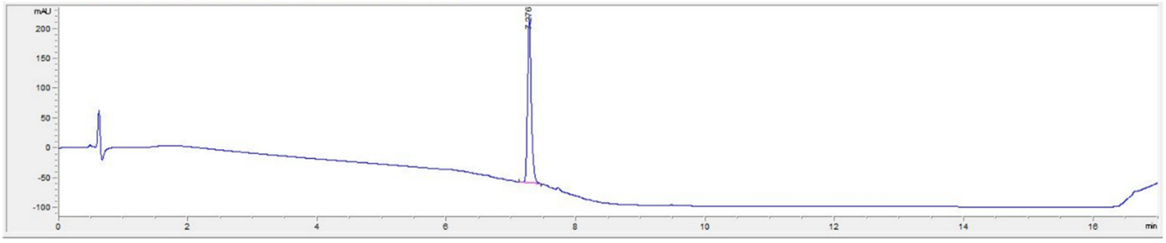

FITC-V26-Pep-8 (>95% purity)

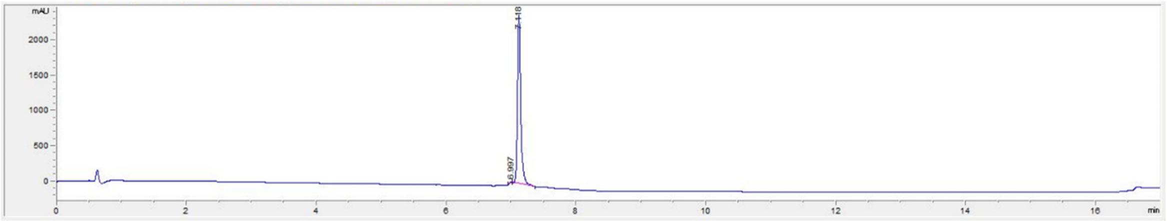

Figure S1: HPLC chromatogram of the purified peptides.

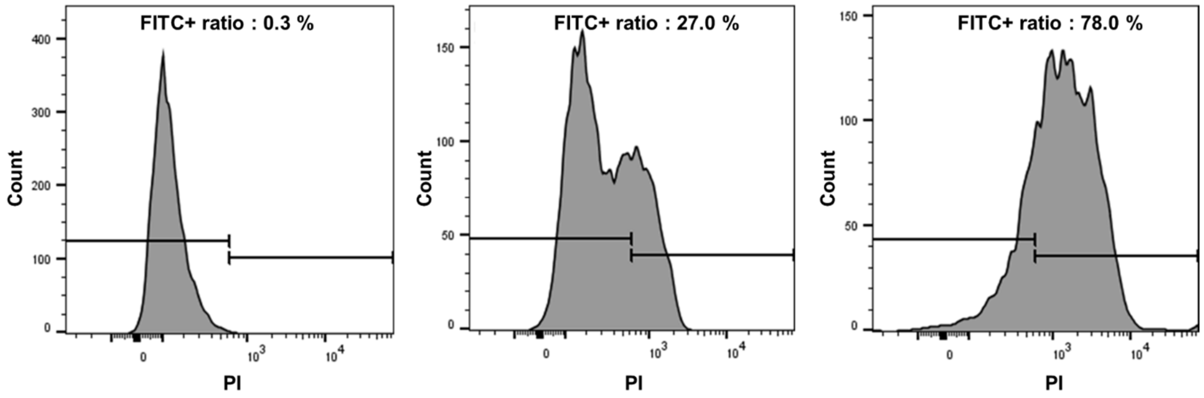

Figure S2: Flow cytometry data of *M. smegmatis* cells obtained from the untreated (negative control, left) group and the FITC-labeled peptide-added groups (linear and stapled forms in the middle and right, respectively).

a

Entry	Peptide	Sequence
7	**V26-SP-7**	DAELAVLR$\mathbf{R_5}$LA$\mathbf{S_5}$
8	**V26-SP-8**	$\mathbf{R_8}$AELAVL$\mathbf{S_5}$ELA
9	**V26-SP-9**	DAELAVL$\mathbf{S_5}$ELA$\mathbf{S_5}$

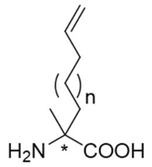

$\mathbf{R_5}$: n = 1, * = *(R)*

$\mathbf{R_8}$: n = 4, * = *(R)*

$\mathbf{S_5}$: n = 1, * = *(S)*

b

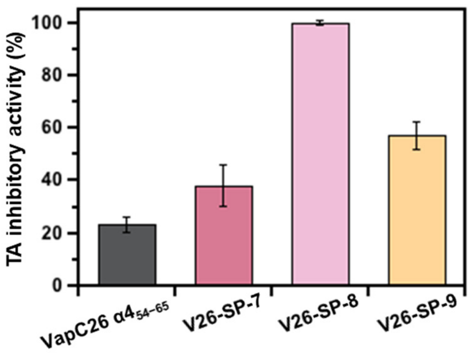

c

V26-SP-7 (>95% purity) **V26-SP-9** (>95% purity)

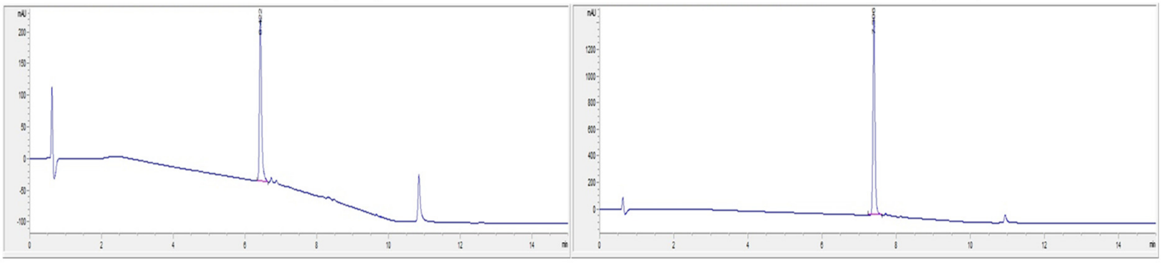

Peptide	Sequence	Chemical Formula	Exact Mass (M+ H⁺)	Exact Mass (M+ Na⁺)	Observed Mas s
V26-SP-7	Ac-Asp-Ala-Glu-Leu-Ala-Val-Leu-Arg-R_5-Leu-Ala-S_5-NH_2	$C_{63}H_{108}N_{16}O_{17}$	1361.8151	1383.7971	1361.8169
V26-SP-9	Ac-Asp-Ala-Glu-Leu-Ala-Val-Leu-S_5-Glu-Leu-Ala-S_5-NH_2	$C_{62}H_{103}N_{13}O_{19}$	1334.7566	1356.7385	1334.7601

Figure S3: Data of the stapled peptides. **(a)** Stapled peptides synthesized based on VapC26 α4_{54-65}. **(b)** *In vitro* ribonuclease activity assay results of stapled peptides. The activity was normalized to that of **V26-SP-8**. The concentration of each peptide was 10 µM. Data are presented as the mean ± SD of three independent replicates. **(c)** HPLC chromatogram and HRMS analysis of two stapled peptides.

Supplementary Table S1: HRMS analysis of peptides.

Peptide	Sequence	Chemical Formula	Exact Mass ($M+H^+$)	Exact Mass ($M+Na^+$)	Observed Mass
V26-SP-8	Ac-R_8-Ala-Glu-Leu-Ala-Val-Leu-S_5-Glu-Leu-Ala-NH_2	$C_{61}H_{104}N_{12}O_{16}$	1261.7766	1283.7586	1283.7585
FITC-V26-SP-8	FITC-Ahx-R_8-Ala-Glu-Leu-Ala-Val-Leu-S_5-Glu-Leu-Ala-NH_2	$C_{86}H_{124}N_{14}O_{21}S$	1721.8859	1743.8678	1743.8689

Supplementary Table S2: Primers used for cloning.

pYUBDuet Vector-Rv0581-Rv0582		
1st PCR	Rv0581-BHI_F	accacagccaggatcc g atggacaagacgacggtc
	Rv0581-HindIII_r	atgcggccgcaagctt tcaccgctcaccgaagcc
2nd PCR	Rv0582-NdeI_F	agaaggagatatacat atgatcatcgacacgagt
	Rv0582-EcoRV-r	gtggccggccgatatc gc cggaatgacggtgaagcg
